# PD-L1 expression in ovarian clear cell carcinoma using the 22C3 pharmDx assay

**DOI:** 10.1186/s13000-024-01510-4

**Published:** 2024-06-15

**Authors:** Yike Gao, Boju Pan, Hongbao Jia, Yang Zhang, Shu Wang, Yuming Wang, Sumei Zhang, Mei Li, Anqi Wang, Xiaoxi Wang, Kun Zhao, Zixin Zhang, Jian Sun, Dan Guo, Zhiyong Liang

**Affiliations:** 1 Department of Pathology, Molecular Pathology Research Center, Peking Union Medical College Hospital, Chinese Academy of Medical Science & Peking Union Medical College, Beijing, China; 2 School of Statistics, Renmin University of China, Beijing, China; 3 Department of Obstetrics and Gynecology, Peking Union Medical College Hospital, Chinese Academy of Medical Sciences & Peking Union Medical College, Beijing, China; 4 National Clinical Research Center for Obstetric & Gynecologic Diseases, Beijing, China; 5 Clinical Biobank, Peking Union Medical College Hospital, Chinese Academy of Medical Sciences & Peking Union Medical College, Beijing, China; 6 Department of Medical Research Center, Peking Union Medical College Hospital, Chinese Academy of Medical Sciences & Peking Union Medical College, Beijing, China

**Keywords:** Ovarian clear cell carcinoma, PD-L1, 22C3, Whole sections

## Abstract

**Background:**

Ovarian clear cell carcinoma (OCCC), well known for its chemoresistance to platinum-based chemotherapy, exhibited a good response in clinical trials of anti–PD-1/PD-L1 inhibitors. By assessing PD-L1 expression, we sought to determine the potential therapeutic benefit of PD-1/PD-L1 inhibitors in OCCC.

**Methods and results:**

The retrospective study included 152 individuals with OCCC between 2019 and 2022 at Peking Union Medical College Hospital. Paired tumors of primary versus recurrent lesions (17 pairs from 15 patients) or primary versus metastatic lesions (11 pairs from 9 patients) were also included. The 22C3 pharmDx assay and whole sections were used for PD-L1 immunohistochemical staining. Pathologists with experience in premarket clinical trials evaluated PD-L1 expression based on various diagnostic criteria (TPS 1%, CPS 1, or CPS 10). The number and percentage of positive PD-L1 cases were 34 (22.4%, TPS ≥ 1%) and 59 (38.8%, CPS ≥ 1), respectively. Thirty-three (21.7%) of the cases had high PD-L1 expression (CPS ≥ 10). Half of the platinum-resistant patients (11/22) were PD-L1 positive (CPS ≥ 1). In addition, positive PD-L1 expression (CPS ≥ 1) was related to clinicopathological characteristics that represented a worse prognosis, such as advanced stages, lymph node metastasis, and distant metastasis (*p* = 0.032, *p* < 0.001 and *p* = 0.003, separately). PD-L1 was expressed equally or more in the recurrent lesion compared with its matched primary lesion.

**Conclusions:**

In conclusion, anti–PD-1/PD-L1 inhibitors are a promising therapeutic choice for OCCC. For evaluation of PD-L1 expression, CPS is more recommended than TPS. Evaluation of recurrent lesion was still suitable and predictive when the primary tumor tissue was not available. Distant metastatic lesions can serve as alternative samples for PD-L1 evaluation, while usage of lymphatic metastatic lesions is not recommended.

**Supplementary Information:**

The online version contains supplementary material available at 10.1186/s13000-024-01510-4.

## Introduction

Despite being the eighth most common cancer in women, ovarian carcinoma (OC) is the most fatal gynecological malignancy [1]. Ovarian clear cell carcinoma (OCCC), a histological subtype of ovarian carcinomas, is widely known for its resistance to platinum-based chemotherapy. Patients with OCCC had a worse prognosis than those with other subtypes of OCs, such as high-grade serous carcinoma, at the same stage [2, 3]. The discovery and verification of novel therapies are urgent.

Immune checkpoint blockade (ICB) against the programmed death-1 (PD-1)/programmed death-ligand 1 (PD-L1) system has recently become a revolutionary treatment for solid tumors. The largest clinical trial using anti–PD-1/PD-L1 treatment for OC patients is KEYNOTE-100. It has been found that patients with OCCC have a higher response rate than those with other OC subtypes [4]. Anti–PD-1/PD-L1 inhibitor use in the clinical therapy of OCCC is therefore promising.

Anti–PD-1/PD-L1 drugs improve the ability of immune cells, particularly T lymphocytes, to specifically kill tumor cells while inflicting little harm to healthy cells [5]. However, not all patients can benefit from this treatment. Given the high cost of therapy with anti–PD-1/PD-L1 drugs, it is reasonable to exclusively treat those individuals who will most likely benefit [6].

PD-L1 expression is currently the most effective validated predictive biomarker for cancer immunotherapy. Nevertheless, even though PD-L1 identification is essential, there are certain challenges with evaluating it via immunohistochemistry.

First, different PD-L1 antibodies are not equal in terms of analytical performance [7]. To date, the Food and Drug Administration (FDA) has approved four companion diagnostic assays for PD-L1 immunohistochemistry. Dako 22C3 has been approved for PD-LI detection in pembrolizumab treatment of several solid tumors, including non-small cell lung cancer, esophageal squamous cell carcinoma and cervical cancer. The other three assays—Ventana SP142, Dako 28 − 8, and Ventana SP263—have been approved for use with different immunotherapeutic drugs and cancer types [8]. The 22C3 assay, out of the four, is the most sensitive for detecting tumor cell expression [9]. Its matching drug pembrolizumab was the first approved and most widely used immunotherapy drug [10, 11]. Thus far, several studies have reported PD-L1 expression in OCCC; however, some of them did not use assays that were approved by the FDA [12–15].

Additionally, spatial and temporal PD-L1 heterogeneity is still an important problem in PD-L1 interpretation [16]. Significant differences in PD-L1 expression have been discovered within tumors [17] and between primary tumors and their metastases or recurrences [18, 19]. A tissue microarray (TMA) containing at least 5 cores of every sample was concordant with the evaluation of whole Sect [20]. However, in previous studies of PD-L1 expression in OCCC, most studies utilized TMAs with 2–3 cores per case, which was not enough to represent the whole tissue Sects [12–14, 21–25]. Chen et al. used whole sections and 22C3 antibodies for PD-L1 scoring, but the study only included 24 patients with OCCC [26].

In this study, we utilized the 22C3 pharmDx assay and whole sections for immunohistochemical staining of PD-L1. We also included paired samples of primary cancers and their metastases or recurrences.

## Materials and methods

### Patient samples and clinical data

The retrospective study included 152 patients diagnosed with OCCC at Peking Union Medical College Hospital (Beijing, China) from 2019 to 2022. No adjuvant therapy, such as radiotherapy or chemotherapy, was performed before surgery. The diagnosis was reconfirmed by 3 experienced pathologists based on WHO guidelines introduced in 2020 [27].

Finally, primary lesions from 152 patients were involved in the evaluation of PD-L1 expression. Clinical information was collected. The 2014 FIGO staging system was used to determine clinical stages. Positive cytology indicated malignant cells in ascites or peritoneal washings. Postoperative resection status was divided into three categories: R0 (no macroscopic residual tumor), R1 (residual lesion ≤ 1 cm), and R2 (residual lesion > 1 cm) [28]. Drug sensitivity was evaluated. Platinum-resistant disease was defined as progression or persistent disease on maintenance therapy or complete remission and relapse within 6 months after completion of platinum-based chemotherapy [12, 29].

To assess the difference in PD-L1 expression between primary and recurring lesions or between primary and metastatic lesions, paired lesions diagnosed from 2015 to 2022 were also included. Ultimately, 17 pairs of primary lesions versus recurrent lesions from 15 patients were included, while another 11 pairs of primary lesions versus metastatic lesions from 9 patients were evaluated.

This study was approved by the Institutional Review Board of Peking Union Medical College Hospital (I-22PJ1120). Informed consent forms were obtained from all patients.

### Immunohistochemical staining

Four-micron paraffin sections were processed by deparaffinization, rehydration and target retrieval (3 in 1) procedures with Dako PT Link (Dako, Glostrup, Denmark). The sections were incubated in Target Retrieval Solution (Low pH, 1x working solution) at 97℃ for 20 min and room temperature Wash Buffer (K8007) for 5 min. The PD-L1 IHC 22C3 pharmDx kit, Code SK006 (Agilent Technologies, Santa Clara), was used on the Dako Autostainer 48 platform (Agilent Technologies, Santa Clara) following the manufacturer’s guidelines. After the staining procedure, slides were counterstained for 5 min with hematoxylin (K8008) and then mounted.

Serial whole sections from the same paraffin block were stained with hematoxylin and eosin (H&E) and the PD-L1 IHC 22C3 pharmDx. The NCI-H226 cell line was utilized as a positive control for immunohistochemistry, while the MCF-7 cell line was employed as a negative control.

### Evaluation of PD-L1 expression

PD-L1 expression was mainly evaluated by 2 experienced pathologists (Y.-K.G. and B.-J.P.) who had taken part in several premarket clinical trials on antiPD-1/PD-L1 therapy. A third pathologist (J.S.) assisted in reviewing the disparate findings of the two pathologists under a multihead microscope.

Both the tumor proportion score (TPS) and combined positive score (CPS) were calculated to determine PD-L1 expression. TPS was defined as the percentage of viable tumor cells showing partial or complete membrane staining at any intensity. CPS was the number of PD-L1 stained cells (tumor cells, lymphocytes, macrophages) divided by the total number of viable tumor cells, multiplied by 100. Only the immune cells infiltrating the invasive tumor and its associated intra- and peri-tumoral stroma were to be scored [30]. It was noteworthy, nevertheless, that metastatic lymph nodes are not ideally suitable for assessing PD-L1 using the CPS system because tumor-infiltrating lymphoid cells can be difficult to distinguish and measure from background lymphoid cells.

Different cutoff values were utilized to determine the expression of PD-L1. Positive PD-L1 expression was defined as TPS 1% or CPS 1 or more of PD-L1 expression. A CPS ≥ 10 was determined as high PD-L1 expression, which could be related to a better reaction to immunotherapy [31].

### Statistical analysis

Statistical analyses were performed with R software, version 4.3.1 (R Foundation for Statistical Computing), and SPSS version 24.0 for Windows (IBM). Pearson’s Chi-squared test and Fisher’s exact test were applied to analyze associations between binary variables, such as PD-L1 staining results or other clinicopathological characteristics. Univariate and multivariable analyses based on progression-free survival (PFS) were performed using Cox regression. The proportional hazards assumption was examined by testing the statistical significance of interactions between follow-up time and exposures. Any differences in PFS between patients with various levels of PD-L1 expression were assessed using Kaplan‒Meier curves. A *P* value < 0.05 was considered to indicate statistical significance.

## Results

### Clinicopathological information and expression of PD-L1

The patients’ age ranged between 25 and 77 years (median 51 years). More than half of the patients were diagnosed with stage I disease (FIGO, 2014). In 40 of 152 (26.3%) patients, malignant cells were found in ascites or peritoneal washings. In 38.8% of cases, tumor rupture occurred during surgery. The majority of patients had optimal debulking (93.4%) and underwent adjuvant chemotherapy (95.4%). Most cases were related to endometriosis (67.8%). Details of clinicopathological characteristics are summarized in Table 1.

**Table 1:** Clinicopathological data of patients with primary ovarian clear cell carcinoma (*n* = 152)

Age	51 (44–57)
Stage	
I	92 (60.5%)
II	25 (16.4%)
III	26 (17.1%)
IV	9 (5.9%)
Bilateral tumors	20 (13.2%)
Tumor size/cm	11.9 (8.8–14.5)
Pelvic metastasis	41 (27.0%)
Lymph node metastasis	14 (9.2%)
Distant metastasis	13 (8.6%)
Positive cytology	40 (26.3%)
Tumor rupture	59 (38.8%)
No residual tumor (R0)	142 (93.4%)
Endometriosis	103 (67.8%)
Thrombosis	25 (16.4%)
Chemotherapy	145 (95.4%)
Recurrence	31 (20.4%)
Chemoresistance (*n* = 123)*	22 (17.9%)
Death	2 (1.3%)
PFS/month	15.5 (7.0-27.8)
OS/month	17.0 (9.0-30.8)
PD-L1 TPS ≥ 1%	34 (22.4%)
PD-L1 CPS ≥ 1	59 (38.8%)
PD-L1 CPS ≥ 10	33 (21.7%)

Continuous variables such as age, tumor size, PFS and OS were presented as median (interquartile range). Bilateral variables were presented as number of cases (percentage)

*Data deficiency due to lack of medical records

**Fig. 1:**
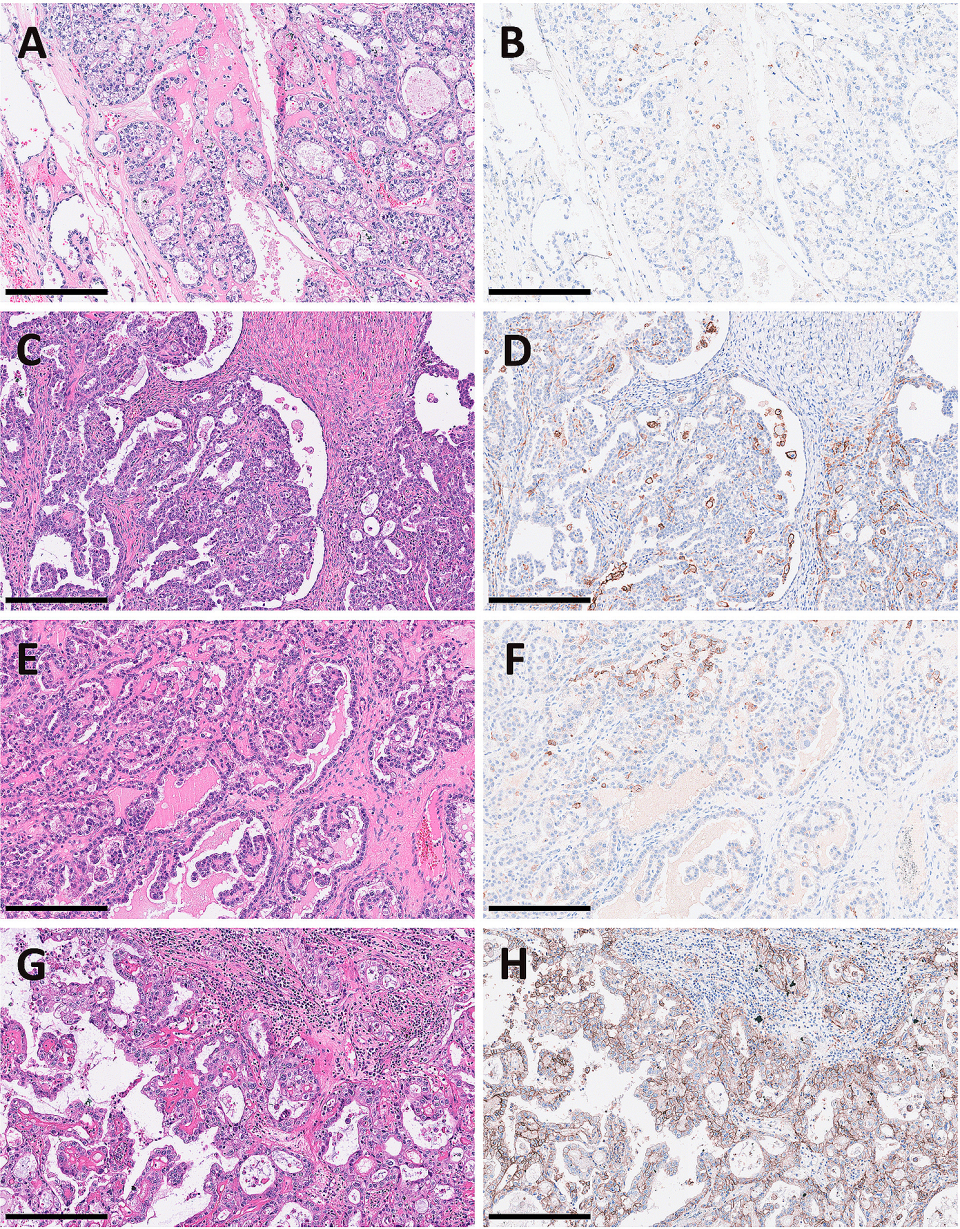
Typical images of OCCCs with different scores of PD-L1 expression Images of OCCCs with hematoxylin and eosin (H&E) staining and matching PD-L1 immunohistochemical results are displayed; different levels of PD-L1 expression are shown; **A&B)** TPS < 1% and CPS < 1; **C&D)** TPS < 1% while CPS ≥ 1; **E&F)** TPS > 1%; **G&H)** CPS ≥ 10; the scale bar is 250 μm

PD-L1 expression was evaluated with three different cutoff levels. Figure 1 displays typical figures. The number and percentage of positive PD-L1 cases with different dividing values of TPS 1% and CPS 1 were 34 (22.4%) and 59 (38.8%), respectively. Thirty-three (21.7%) of the cases had high PD-L1 expression (CPS ≥ 10). Among 123 patients with sufficient follow-up time to determine drug sensitivity, 22 were platinum-resistant. 27.3% or 50% of the platinum-resistant patients were PD-L1 positive based on different dividing values. Five patients (22.7%) that were resistant to platinum had high PD-L1 expression (CPS ≥ 10).

Calculations were made to determine the relationship between PD-L1 expression and clinicopathological data (Table S1). Whether based on TPS or CPS, positive PD-L1 was statistically linked to lymph node metastasis (*p* = 0.023 or *p* < 0.001). In addition, positive PD-L1 expression (CPS ≥ 1) was also related to advanced disease and distant metastasis (*p* = 0.032 and *p* = 0.003, separately).

### Analysis of prognostic factors

**Fig. 2:**
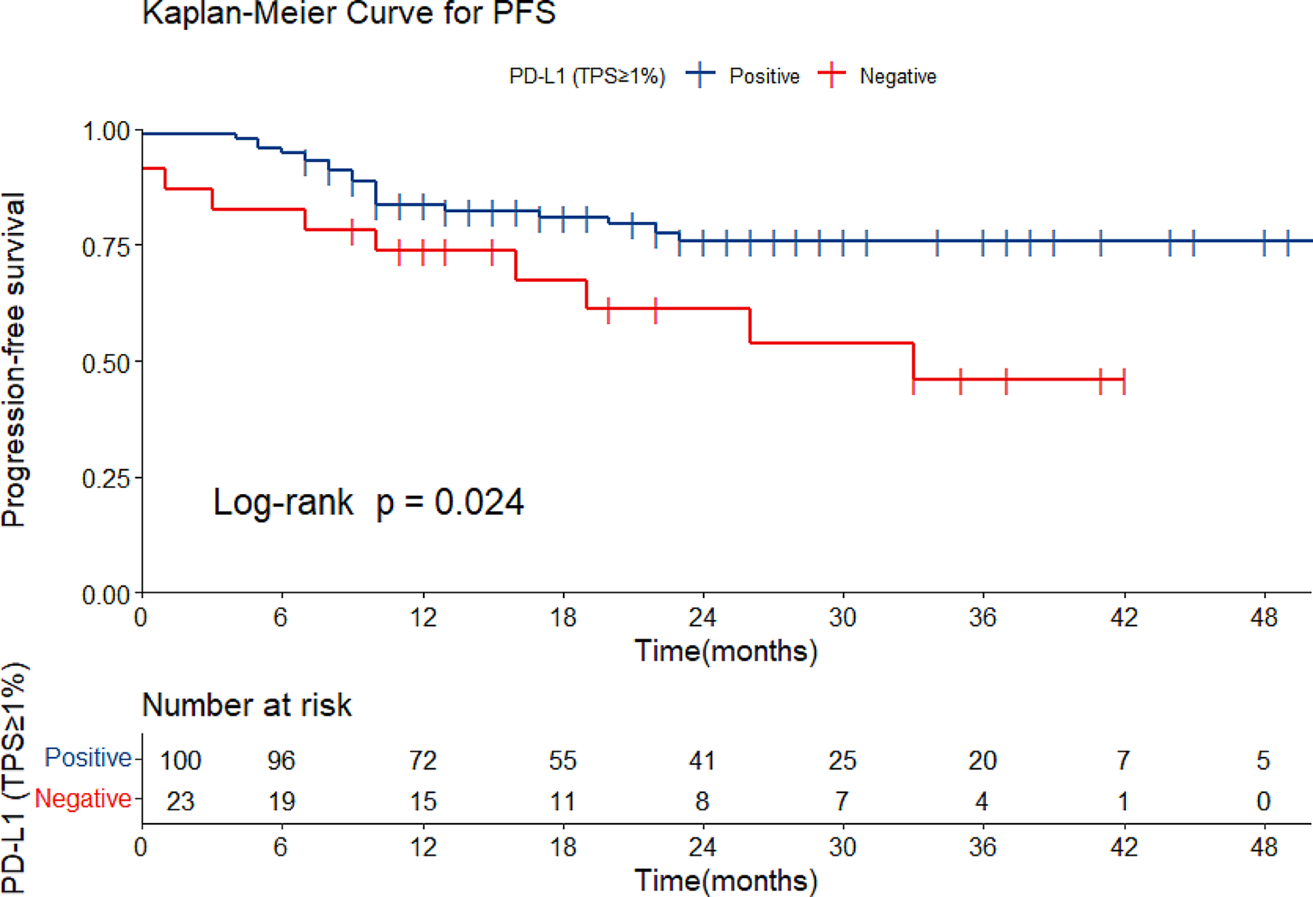
Progression-free survival curves of patients with positive or negative PD-L1 expression (*n* = 123) The cutoff value of PD-L1 expression was TPS 1%

Prognostic analyses based on Cox regression were carried out. In the univariate analysis (Table S2), the hazard ratio for recurrence was 2.31 (95% CI 1.09–4.92, *p* = 0.029) in patients with positive PD-L1 expression (TPS ≥ 1%). Kaplan‒Meier curves were also drawn to show relationships between PD-L1 expression and PFS (Fig. 2). In addition, a poor prognosis was also associated with advanced stages, bilateral tumors, pelvic implants, lymph node metastases, distant metastasis, positive cytology, and residual tumor (Cox regression, univariate analysis).

**Fig. 3:**
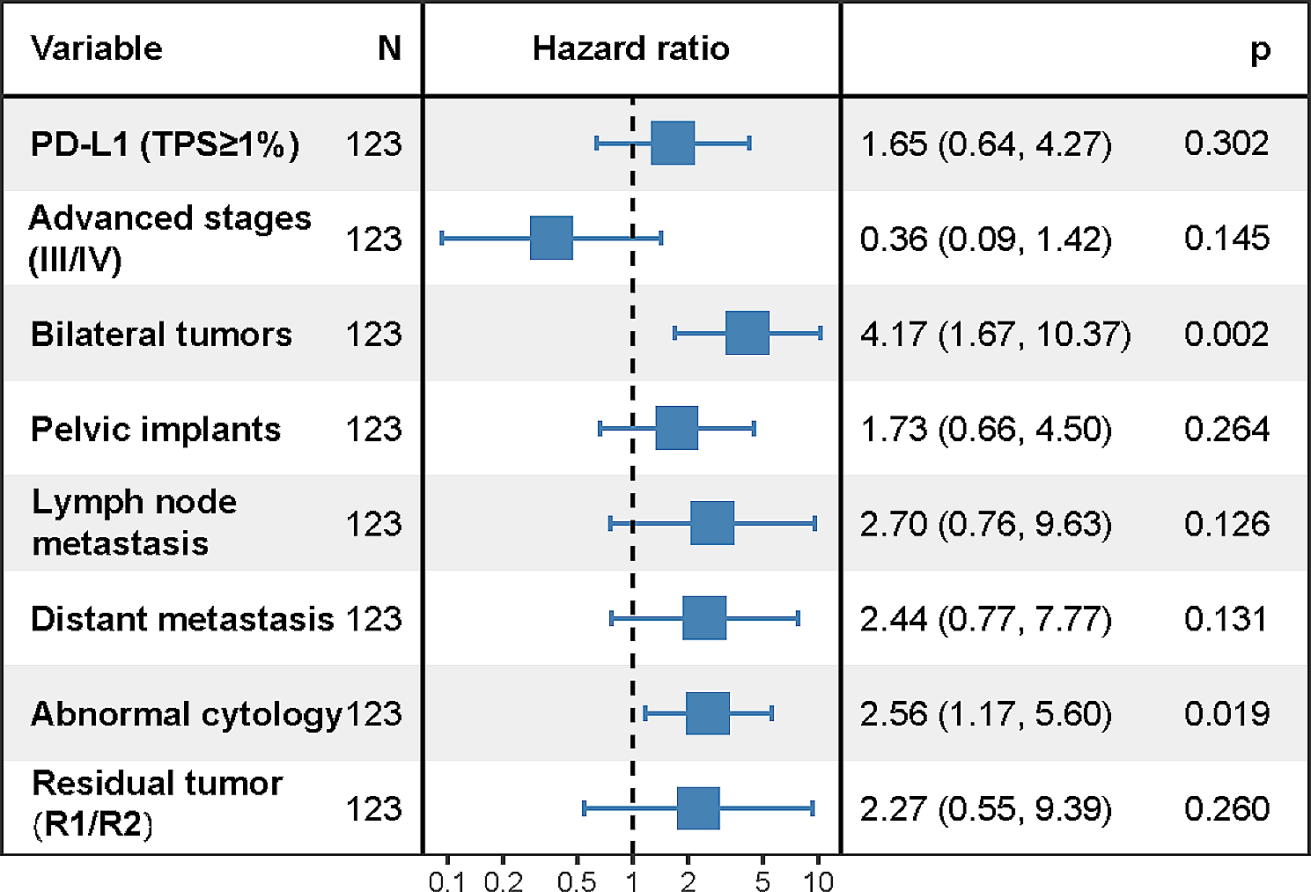
Multivariate analysis of factors associated with progression-free survival by Cox regression (*n* = 123)

Bilateral tumors and positive cytology were still linked to a poor prognosis in the multivariate analysis (Fig. 3), with hazard ratios of 4.17 (95% CI 1.67–10.37, *p* = 0.002) and 2.56 (95% CI 1.17–5.60, *p* = 0.019), respectively.

### Difference in PD-L1 expression between primary and recurrent lesions

The PD-L1 expression status of the 17 pairs of primary and recurrent lesions is listed in Table 2. Among the 15 included patients, four underwent immunotherapy and subsequentially experienced partial or complete response. However, determining the correlation between PD-L1 expression and treatment response is difficult due to the variation in the treatment courses and antibodies of anti–PD-L1 therapy received by the four patients. Patient R12 received immunotherapy before the submission of the recurrent lesion, while the other three patients were given treatment after surgery at the recurrent sites.

**Table 2:** PD-L1 expression of matched primary lesions and recurrent lesions

Patient number	Pair number	FIGO Stage	PFS/ months	Drug sensitivity	Primary lesion		Recurrent lesion	
					TPS/%	CPS	TPS/%	CPS
R1	R1	IIIC	17	PS	5	5	50	50
R1	R2	/	/	/	/	/	20	25
R2	R3	IA	58	PS	0	< 1	20	30
R3*	R4	IC	45	PS	< 1	< 1	2	3
R4	R5	IIB	13	PS	0	0	1	1
R5	R6	IIIB	36	PS	0	5	5	10
R6	R7	IC	9	PR	0	0	0	0
R7	R8	IIIA	29	PS	< 1	< 1	5	40
R8*	R9	IC	25	PS	0	0	< 1	2
R9	R10	IC	10	PR	0	0	0	0
R10*	R11	IIIC	9	PR	5	5	8	8
R11*	R12	IC	19	PS	1	1	20	25
R11	R13	/	/	/	/	/	30	30
R12*	R14	IIB	22	PS	0	0	< 1	10
R13	R15	IV	6	PR	< 1	5	0	10
R14	R16	IIIA	23	PS	< 1	< 1	5	5
R15	R17	IIB	13	PS	0	0	< 1	1

Pairs were sorted by the time of the first operation. PS: platinum-sensitive; PR: platinum-resistant

*Patient R3 received neoadjuvant therapy. Patient R8, R10, R11 and R12 all went through immunotherapy. Patient R12 received immunotherapy before the surgery of the recurrent lesion, while others received immunotherapy afterwards

When compared to original tumors, a greater numerical value of TPS or CPS was seen in the majority of recurring tumors (15/17, 88.2%). In the other two pairs (pairs R7 and R10), both primary and recurrent tumors lacked PD-L1 expression. In addition, the CPS of all primary lesions was lower than 10, while more than half (9/17) of the recurrent lesions reached a CPS of 10.

We found that in a specific group of patients, PD-L1 was expressed in recurrent lesions but not in original lesions (6/15 based on TPS and 8/15 based on CPS). These patients were all sensitive to platinum-based therapy, but some patients suffered from platinum resistance with PD-L1 expression status unchanged between primary and recurrent lesions. In addition, when the dividing value was TPS 1%, patients with increased PD-L1 results in recurring lesions (*n* = 6) had a statistically longer PFS (Mann‒Whitney U test, Z=-2.326, *p* = 0.015), compared to patients with consistently negative PD-L1 expression (*n* = 6). Similar conclusion was reached when the cut-off value changed to CPS 1 (*n* = 8 vs. *n* = 2, Mann‒Whitney U test, Z=-2.095, *p* = 0.044). Remarkably, 3/6 of the patients with increased PD-L1 results were diagnosed in late stages, compared to 1/6 of the patients with consistently negative PD-L1 expression (TPS 1%). Similar results were found with the dividing value CPS 1 (2/6 vs. 0/2).

### Differences in PD-L1 expression between primary and metastatic lesions

The PD-L1 expression of 11 pairs of primary and metastatic lesions is listed in Table S3. For the lymph node metastatic site, only its TPS value was evaluated. When the cutoff value was TPS 1%, most pairs of primary and metastatic lesions had similar PD-L1 expression (7/11, 63.6%). The PD-L1 expression status was consistent between primary and distant metastatic sites (Pair M6).

Surprisingly, in patient M5 (Figure S1), we found that both the primary lesion and the liver-metastatic site exhibited TPS ≥ 1% and CPS ≥ 10. However, in the metastatic site of the retroperitoneal lymph node, TPS was less than 1%.

## Discussion

Our study is thus far the largest study to provide an analysis of PD-L1 expression in OCCC by evaluating primary lesions. We are the first to evaluate the difference in PD-L1 expression in paired samples from the same patient to compare primary tumors and recurrent or metastatic lesions. We also compared PD-L1 expression between patients with different levels of chemosensitivity.

The PD-L1 IHC 22C3 pharmDx kit was applied. It is the most sensitive PD-L1 antibody and is closely related to clinical medicine. We used whole sections instead of TMAs to ensure the accuracy of PD-L1 evaluation. Our standard of PD-L1 evaluation was the same as that in clinical trials, and our judging pathologists were experienced in PD-L1 evaluation involved in clinical trials. We applied three different cutoff values of PD-L1 expression in this study, including TPS 1%, CPS 1 and CPS 10. To determine the three dividing values, we referred to the recommended thresholds in other carcinomas and the results of OCCC clinical trials. PD-L1 expression in non-small cell lung carcinoma is determined by TPS 1%. In the largest PD-L1 clinical trial of ovarian cancer (KEYNOTE-100), CPS 1 or CPS 10 was applied as the cutoff value. Additionally, the response rate was higher in the group with CPS 10 [4, 31].

In our study, 22.4 ∼ 38.8% of OCCC patients were PD-L1 positive based on different diagnostic criteria. The results were similar to those of most previous studies with a positive percentage of PD-L1 at 16.7 ∼ 33.3% [12–14, 21–23, 26, 32]. However, in a recent Japanese study, up to 86.4% of the cases were PD-L1 positive [15]. Since the study applied different antibodies from other studies (Clone 27A2, MBL), it was hard to tell whether there was true difference between different groups of patients. In addition, 21.7% of OCCCs have high PD-L1 expression (CPS ≥ 10), which is related to a high response rate for anti–PD-1/PD-L1 therapy. The proportion of PD-L1 positive cases was also quite considerable in patients who were platinum-resistant (50.0% when the cutoff value was CPS 1). Given that immunotherapy response in tumors is predicted by PD-L1 expression, it is reasonable to assume that anti–PD-1/PD-L1 therapy may be effective in the treatment of OCCC.

The effect of PD-L1 expression on prognosis was controversial based on previous studies [12, 22, 26, 32]. In our study, PD-L1 TPS ≥ 1% was significantly related to shorter PFS in the univariate analysis. However, there was no statistical significance after adjustment. When the cutoff value was CPS 1, high PD-L1 expression was related to clinicopathological characteristics that represented a worse prognosis, such as advanced stages or metastasis to lymph nodes and distant sites.

Paired samples of primary lesions and recurrent or metastatic lesions were included in our study. Li et al. compared PD-L1 expression in primary and recurrent tumors [32], but the tumors were from different patients and thus lacked comparability. Concerning metastatic lesions, metastatic modes of OCCC include lymphatic, hematogenous and implantation metastasis. Parvathareddy et al. focused on implantation and studied the differences between primary tumors and peritoneal metastatic tumors [14]. In our study, we paid close attention to lymphatic or hematogenous metastasis by including matched metastatic lesions to lymph nodes or distant organs.

Remarkably, in contrast to original tumors, we only observed increase and not a reduction in PD-L1 expression in recurrent cancers. In a comparative study of PD-L1 expression in matched primary and recurrent glioma, PD-L1 expression also showed an upward trend in recurrences [18]. According to Fernandez, the 22C3 epitope is not stable over time, and the signal is more likely to be lost in older tissue than in fresh tissue [33], which may also contribute to our findings. In conclusion, evaluation of recurrent lesion was still suitable and predictive when the primary tumor tissue was not available. Regarding the explanation of the mechanism, the PD-L1 pathway was possibly more activated in recurrent lesions than in primary lesions. Since PD-L1 participates in immune escape, increased expression of PD-L1 means that the tumor is more immune-tolerant.

Additionally, similar to a previous study in glioma [18], we also found that for those patients whose primary lesion was PD-L1 negative, gain of PD-L1 expression in the recurrent site was related with better prognosis (*p* < 0.015 or *p* = 0.044). More unexpectedly, more patients with elevated PD-L1 results had late-stage diagnoses, which should lead to poorer prognosis. In our cohort, patients whose PD-L1 expression was higher in recurrent tumors than in primary tumors were all platinum-sensitive, which may partly explain their better prognosis. However, limited by the small sample size in this study, the relationship between the increase in PD-L1 and the response to platinum-based therapy is worth further investigation.

Furthermore, whether the increase in PD-L1 expression in recurrent lesions is related to a better response to immunotherapy is still puzzling. Only four patients in our cohort underwent immunotherapy and were treated with different immune checkpoint inhibitors. We noticed that the CPS of these patients’ recurrent sites was higher than 1, while the TPS of patients R8 and R12 was lower than 1%. Complete or partial response was observed in the four patients. As a result, the utilization of CPS may be a better choice when evaluating PD-L1 expression in OCCC.

Selecting the number and location of lesions to assess following the initial surgery is challenging due to the notable spatial variability of PD-L1 expression [19, 34]. We compared PD-L1 expression between primary tumors and their concurrent metastases. As the lymph node metastatic lesion may differ significantly from the initial lesion, it would not be a viable candidate for PD-L1 screening. In contrast, distant metastatic tumors showed PD-L1 expression consistent with primary tumors and could provide an alternative sample for PD-L1 evaluation.

Our study should be interpreted within its limitations. The small sample size of platinum-resistant patients or paired lesions underscores the necessity for additional study. Furthermore, due to the limited use of anti–PD-L1 therapy as the second-line chemotherapy for OCCC, there is a lack of treatment data. To determine the therapeutic impact of anti–PD-1/PD-L1 therapy, more research is needed.

In conclusion, we view the anti–PD-1/PD-L1 inhibitor as a viable therapeutic option for OCCC after assessing the expression of PD-L1 by immunohistochemical staining. PD-L1 expression of the recurrent tumor was higher or equal to the primary tumor. Evaluation of recurrent lesion can serve as an alternative when primary lesion was not available. Considering the response of immunotherapy, using of CPS might be better than TPS. These findings provide valuable experience for PD-L1 evaluation in OCCC.

### Electronic supplementary material

Below is the link to the electronic supplementary material.

Supplementary Material 1

## Data Availability

No datasets were generated or analysed during the current study.
