# Supplementary material for: PD-L1 expression in ovarian clear cell carcinoma using the 22C3 pharmDx assay
Source: Diagn Pathol. 2024 Jun 15;19:82. doi: 10.1186/s13000-024-01510-4 (PMC11179196; doi:10.1186/s13000-024-01510-4)
Supplement: Supplementary file 1 — Supplementary Material 1 [file 13000_2024_1510_MOESM1_ESM.docx]

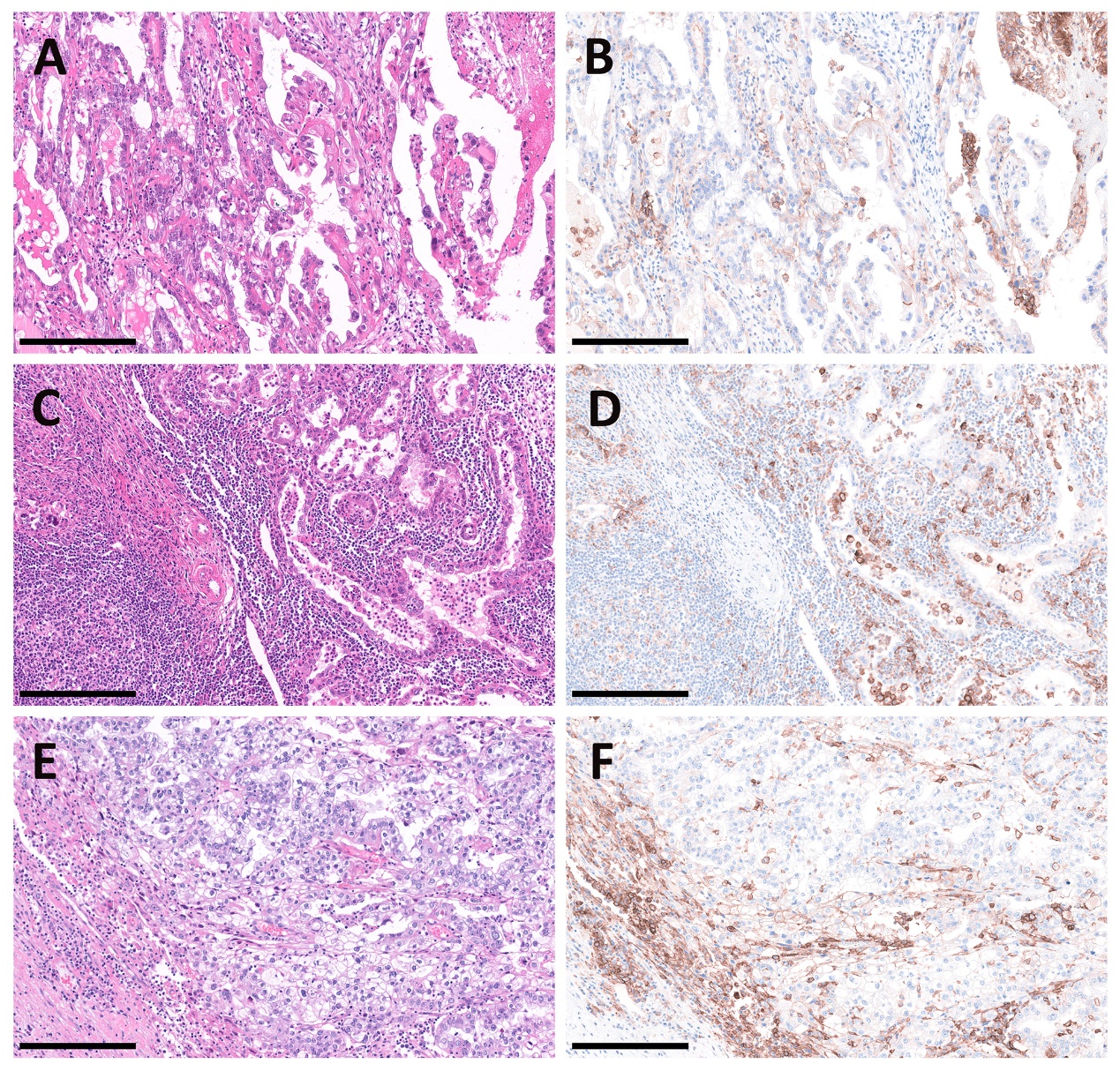


**Figure S1. Special case (patient M5) with lymphatic metastasis and distant metastasis (liver)**

Images include hematoxylin and eosin (H&E) and immunohistochemical results of PD-L1; **A&B)** the primary tumor; **C&D)** metastatic site in retroperitoneal lymph nodes; **E&F)** metastatic site in the liver; the scale bar is 250 μm

Table S1. Relationships between clinicopathological characteristics and PD-L1 expression under different cutoff values (n=152)

|  | TPS 1% | | | CPS 1 | | | CPS 10 | | |
| --- | --- | --- | --- | --- | --- | --- | --- | --- | --- |
|  | Pos | Neg | P value | Pos | Neg | P value | High | Low | P value |
| Stage | | | | | | | | | |
| I/II | 24 | 93 | 0.315 | 40 | 77 | 0.032^a^ | 24 | 93 | 0.513 |
| III/IV | 10 | 25 |  | 19 | 16 |  | 9 | 26 |  |
| Location | | | | | | | | | |
| Unilateral | 30 | 102 | 1.000 | 50 | 82 | 0.543 | 28 | 104 | 0.927 |
| Bilateral | 4 | 16 |  | 9 | 11 |  | 5 | 15 |  |
| Lymph node metastasis | | | | | | | | | |
| With | 7 | 7 | 0.023^a^ | 12 | 2 | <0.001^a^ | 6 | 8 | 0.094 |
| W/o | 27 | 111 |  | 47 | 91 |  | 27 | 111 |  |
| Distant metastasis | | | | | | | | | |
| With | 4 | 9 | 0.680 | 10 | 3 | 0.003^a^ | 6 | 7 | 0.060 |
| W/o | 30 | 109 |  | 49 | 90 |  | 27 | 112 |  |
| Cytology | | | | | | | | | |
| Normal | 7 | 33 | 0.389 | 13 | 27 | 0.340 | 28 | 84 | 0.100 |
| Abnormal | 27 | 85 |  | 46 | 66 |  | 5 | 35 |  |
| Tumor rupture | | | | | | | | | |
| With | 9 | 50 | 0.094 | 21 | 38 | 0.516 | 12 | 47 | 0.744 |
| W/o | 25 | 68 |  | 38 | 55 |  | 21 | 72 |  |
| Endometriosis | | | | | | | | | |
| With | 22 | 81 | 0.665 | 38 | 65 | 0.481 | 19 | 84 | 0.157 |
| W/o | 12 | 37 |  | 21 | 28 |  | 14 | 35 |  |
| Thrombosis | | | | | | | | | |
| With | 5 | 21 | 0.673 | 9 | 17 | 0.629 | 5 | 21 | 0.736 |
| W/o | 29 | 97 |  | 50 | 76 |  | 28 | 98 |  |
| Recurrence | | | | | | | | | |
| With | 10 | 21 | 0.139 | 15 | 16 | 0.220 | 8 | 23 | 0.535 |
| W/o | 24 | 97 |  | 44 | 77 |  | 25 | 96 |  |
| Drug sensitivity (n=123)* | | | | | | | | | |
| PS | 17 | 84 | 0.403 | 32 | 69 | 0.103 | 18 | 83 | 0.816 |
| PR | 6 | 16 |  | 11 | 11 |  | 5 | 17 |  |
| Death | | | | | | | | | |
| With | 0 | 2 | 1.000 | 1 | 1 | 1.000 | 0 | 2 | 1.000 |
| W/o | 34 | 116 |  | 58 | 92 |  | 33 | 117 |  |

a: with statistical significance

*Data deficiency due to lack of medical records

Pos: positive; Neg: negative; W/o: without; PS: platinum-sensitive; PR: platinum-resistant

Table S2 Prognostic value of clinicopathological parameters in OCCC (PFS, n=123, univariate) *

|  | HR | 95% CI | P value |
| --- | --- | --- | --- |
| Age | 0.97 | 0.94 - 1.01 | 0.129 |
| PD-L1 TPS≥1% | 2.31 | 1.09 - 4.92 | 0.029^a^ |
| PD-L1 CPS≥1 | 1.93 | 0.96 - 3.91 | 0.067 |
| PD-L1 CPS≥10 | 1.60 | 0.71 - 3.57 | 0.256 |
| Advanced stages (III/IV) | 3.03 | 1.49 - 6.17 | 0.002^a^ |
| Bilateral tumors | 4.62 | 2.20 - 9.69 | <0.001^a^ |
| Tumor size | 0.98 | 0.90 - 1.06 | 0.569 |
| Pelvic metastasis | 3.08 | 1.52 - 6.23 | 0.002^a^ |
| Lymph node metastasis | 4.18 | 1.86 - 9.36 | 0.001^a^ |
| Distant metastasis | 4.89 | 2.00 - 11.98 | 0.001^a^ |
| Abnormal cytology | 3.11 | 1.54 - 6.31 | 0.002^a^ |
| Tumor rupture | 0.99 | 0.49 - 2.03 | 0.988 |
| Endometriosis | 0.74 | 0.36 - 1.52 | 0.410 |
| Thrombosis | 0.97 | 0.40 - 2.37 | 0.946 |
| Residual tumor (R1/R2) | 5.29 | 1.81 - 15.51 | 0.002^a^ |
| Chemotherapy | 0.23 | 0.03 - 1.71 | 0.152 |
| Platinum-resistance | 1.29E+10 | 0 - Inf | 0.997 |

a: with statistical significance.

*Progression free survival (PFS) was used in prognostic analysis. Some cases were not involved because of incomplete medical records.

Table S3. PD-L1 expression of matched primary lesions and metastatic lesions

| Patient number | Pair number | Metastatic sites | PFS/months | Drug sensitivity | Primary lesion | | Metastatic lesion | |
| --- | --- | --- | --- | --- | --- | --- | --- | --- |
|  |  |  |  |  | TPS/% | CPS | TPS/% | CPS |
| M1 | M1 | Paraaortic LN | 35 | PS | 15 | 30 | 40 | / |
| M2 | M2 | Internal iliac LN | 15 | PS | 1 | 1 | <1 | / |
| M3 | M3 | Paraaortic LN | 8 | PR | 0 | <1 | 25 | / |
| M4 | M4 | Internal iliac LN | 10 | PR | 1 | 1 | 0 | / |
| M5 | M5 | Paraaortic LN | 29 | PS | 20 | 25 | <1 | / |
| M5 | M6 | Liver | / | / | / | / | 15 | 30 |
| M6 | M7 | Internal iliac LN | 3 | PS | 90 | 100 | 70 | / |
| M7 | M8 | External iliac LN | 11 | PS | 0 | 2 | 0 | / |
| M8 | M9 | Internal iliac LN | 8 | PS | <1 | 10 | 0 | / |
| M8 | M10 | Paraaortic LN | / | / | / | / | <1 | / |
| M9 | M11 | External iliac LN | 4 | PS | 1 | 20 | 3 | / |

Pairs were sorted by the time of the first operation; PS: platinum-sensitive; PR: platinum-resistant

LN: lymph node; evaluation of CPS is not suitable for lymphatic metastatic sites
